# Postmenopausal osteoporosis increases periodontal inflammation and the pathogenicity of the oral microbiota in a rat model

**DOI:** 10.1080/20002297.2025.2554381

**Published:** 2025-09-01

**Authors:** Miao Lu, Yanan Zhang, Yang Zhang, Xulei Yuan, Tingwei Zhang, Jinlin Song

**Affiliations:** Chongqing Key Laboratory for Oral Diseases and Biomedical Sciences, Chongqing Municipal Key Laboratory for Oral Biomedical Engineering of Higher Education, and Stomatological Hospital of Chongqing Medical University, Chongqing, China

**Keywords:** Postmenopausal osteoporosis, inflammation, oral microbiota, periodontitis, ovariectomy, 16S rRNA

## Abstract

**Objectives:**

This study aims to explore the mechanisms of the detrimental effects of postmenopausal osteoporosis (PMO) on periodontitis.

**Methods:**

An ovariectomized (OVX) rat model was established to investigate the effects of PMO on alveolar bone homeostasis and periodontal inflammation. Chlorhexidine digluconate (CHX) was administered to rats with OVX – periodontitis to ascertain the involvement of the oral microbiota in the influence of PMO on periodontitis. Finally, oral microbiota transplantation was conducted to examine the oral microbiota’s pathogenicity.

**Results:**

OVX rats exhibited increased periodontal trabecular bone resorption and inflammation. In addition, depletion of the oral microbiota by CHX decreased the alveolar bone destruction in OVX – periodontitis rats. Furthermore, 16S rRNA gene sequencing demonstrated that PMO changes the composition of the oral microbiota. Finally, oral microbiota transplantation indicated that PMO enhanced the oral microbiota’s pathogenicity.

**Conclusion:**

PMO detrimentally affects periodontitis by increasing periodontal inflammation and the pathogenicity of the oral microbiota, which provides a mechanistic understanding of how PMO affects periodontitis and highlights the necessity of more regular monitoring of the oral microbiota in PMO patients.

## Introduction

Periodontitis is a chronic inflammatory disease, featured by alveolar bone loss and the primary cause of tooth loss in adults. According to the FDI World Dental Federation’s report, periodontal disease affects up to 50% of the global population [1]. It has been recognized that periodontitis involves complex dynamic interactions between pathogenic microorganisms and destructive immune responses [2]. Furthermore, many risk factors would accelerate the development of periodontitis, such as diabetes, smoking, obesity, and postmenopausal osteoporosis (PMO) [3].

PMO, caused by estrogen deficiency, is a bone metabolic disorder, and can lead to osteoporotic fractures. About 30–55% of postmenopausal women suffer from osteoporosis [4]. Women with PMO are more susceptible to alveolar bone loss [5]. Rats with estrogen deficiency and periodontitis also exhibited more severe periodontal bone destruction than rats with periodontitis alone [6]. However, the underlying mechanism of the detrimental effects of PMO on periodontitis is not entirely defined yet.

Both PMO and periodontitis share a close relationship with inflammation. Estrogen deficiency would cause the body in a chronic low-grade inflammatory state by altering the levels of inflammatory cytokines and the morphology of immune cells [7]. Researchers have shown that the lack of estrogen increases the expression of inflammatory cytokines, such as PGE2, TNF*α*, M-CSF IL-1, and IL-6 [8,9]. The pro-inflammatory cytokines could work on the osteoblasts and osteoclasts to regulate bone homeostasis. Therefore, it is highly likely that PMO increases periodontal inflammation to assist in the progression of periodontitis.

In addition to inflammation, the oral microbiota is also crucial in alveolar bone metabolism. Polymicrobial synergy and dysbiosis could trigger a dysregulated and destructive host response that aggravates alveolar bone loss. Studies have suggested that oral microbiota played pivotal roles in the effect of some systemic diseases on periodontitis. Experimental arthritis triggers periodontitis depending on oral microbiota [10]. Xiao et al. also demonstrated that diabetes could change the oral microbiota composition and increase its pathogenicity, thus accelerating alveolar bone resorption [11]. Therefore, it is crucial to thoroughly investigate the contribution of the oral microbiota to the adverse effects of PMO on alveolar bone loss.

This study used rat models to examine how PMO negatively affects alveolar bone homeostasis by increasing periodontal inflammation and oral microbiota’s pathogenicity. The results provide a mechanistic understanding of how PMO raises the risk and severity of periodontitis.

## Materials and methods

### Experimental design

All the animal experiments of this study were approved by the Ethics Committee of the College of Stomatology, Chongqing Medical University ((Ethics Number: 2022(154))). Ten-week-old female Sprague-Dawley rats were raised in a specific pathogen-free environment with ad libitum access to food and water. A one-week adaptation period was allowed, and the general condition of the animals was monitored daily.

For the first set of experiments, 16 rats were randomly divided into 2 groups (*n* = 8): OVX group and sham group. Rats in the OVX group underwent bilateral ovariectomy, whereas the sham group rats underwent sham surgery. Eight weeks later, all rats were killed in a CO_2_ euthanasia chamber.

For the second set of experiments, 12 rats were randomly divided into 2 groups (*n* = 6): OVX + ligation + chlorhexidine digluconate (OVX + Lig + CHX) and OVX + ligation + PBS (OVX + Lig + PBS). All rats underwent bilateral ovariectomy. Three weeks after ovariectomy, the maxillary second molars of the rats were ligated with a 3–0 silk suture for periodontitis. One week after ligation, the OVX + Lig + CHX group rats received the topical delivery of 0.12% CHX (Macklin, Shanghai, China) daily in the oral cavity, plus 2% carboxymethylcellulose sodium (CMC, Solarbio, Beijing, China). Meanwhile, the silk ligatures were changed daily in the OVX + Lig + CHX group. The rats in the OVX + Lig + PBS group received a topical delivery of PBS daily along with 2% CMC. The rats were killed after four weeks.

Finally, the third set of experiments was conducted. First, 12 rats were randomly divided into 2 groups: 6 underwent bilateral ovariectomy and 6 underwent sham surgery. Eight weeks after the OVX/sham operation, the oral microbiota from the OVX and sham rats were collected, respectively, as previously described in a study [11]. Three weeks after the ovariectomy/sham operation, another 18 rats were purchased and randomly divided into 3 groups (*n* = 6): pseudo-germ-free (PGF) + Lig + transplantation of bacteria from the OVX rats group (bacteria from OVX), PGF + Lig + the transplantation of bacteria from the sham rats group (bacteria from sham), and PGF + Lig + antibiotic cocktails (Abx) group (PGF). Abx (100 mg/kg of ampicillin, 100 mg/kg of metronidazole, 100 mg/kg of neomycin, and 50 mg/kg of vancomycin, all from Solarbio, Beijing, China) were dissolved in drinking water and administered ad libitum to rats for four weeks to establish the PGF rats [12,13]. Then, the rats were ligated for periodontitis. Meanwhile, bacteria from the OVX group rats were inoculated with 200 *μ*L of the oral bacterial sample from the OVX rats, along with 2% CMC, twice with a one-day interval. Bacteria from the sham group rats were inoculated with 200 *μ*L of the oral bacterial sample from the sham rats, along with 2% CMC, twice with a one-day interval. The PGF group rats were maintained on Abx in drinking water. The rats were killed five weeks after the oral bacteria transplant.

### Micro-computed tomography (micro-CT) analysis

The fixed maxillae were scanned with a micro-CT (*μ*CT40; SCANCO Medical, Switzerland) at the voxel resolution of 15 *μ*m. The following scanner settings were used for scans: X-ray source current of 114 *µ*A and voltage of 70 kVp. Three-dimensional reconstructions were generated, and the mesial and distal cementoenamel junction and alveolar bone crest (CEJ – ABC) distances were measured. The regions of interest (ROI) were defined for the histomorphometric analysis of trabecular bone. The 31st transverse image below the root furcation of the second molar was chosen as the beginning of the ROI (circular with a radius of 0.2 mm). The 61st and 91st transverse images (circle with a radius of 0.3 mm) below the root furcation were also selected. The ROI was obtained after morphing these images. Next, the parameters of the bone volume per tissue volume (BV/TV), trabecular separation (Tb.Sp), trabecular thickness (Tb.Th), and trabecular number (Tb.N) at the ROI were calculated.

### Paraffin sections

The maxillae were decalcified and dehydrated for paraffin embedding. Then, the samples were coronally incised in a slice of 5 *μ*m for subsequent histologic analyses.

### Hematoxylin and eosin (H&E) staining

Based on the manufacturer’s instructions, H&E staining was performed using a defined kit (Solarbio, Beijing, China). The slice images were acquired with an Olympus B×41microscope. Finally, the CEJ – ABC distance at the interproximal sites between the first and second molar was measured.

### Tartrate‐resistant acid phosphatase (TRAP) staining

Periodontal TRAP^+^ cells were stained with a TRAP staining solution and counterstained with methyl green (Vector, CA, USA) [14]. TRAP^+^ giant multinucleated cells were defined as osteoclast-like cells. The osteoclast-like cells were photographed with the Olympus B×41microscope and counted by Image-Pro Plus (IPP; Media Cybernetics, USA).

### Immunohistochemical (IHC) analyses

IHC staining was conducted with a Streptavidin-Peroxidase kit (Bioss Antibodies, Beijing, China). The paraffin sections were antigen repaired in sodium citrate buffer, blocked with goat serum, and then incubated with primary antibodies against OPG (1:250; Bioss Antibodies, bs-0431 R), RANKL (1:300; Bioss Antibodies, bs-0747 R), IL-6 (1:300; Bioss Antibodies, bs-0782 R), IL-17A (1:200; Bioss Antibodies, bs-1183 R), iNOS (1:250; Bioss Antibodies, bs-0162 R), and TNF*α* (1:300; Bioss Antibodies, bs -10,802 R). Subsequently, the slides were cultured with secondary antibodies and colored with diamino-benzidine (ZSJQ-BIO, Beijing, China). Finally, all the slices were counterstained with hematoxylin. The intensity of positive staining areas was determined according to the mean integrated option density (IOD). The images of all stained slides were captured with the Olympus B×41microscope and counted by IPP.

### Quantitative real-time polymerase chain reaction (qRT-PCR)

The gingival tissue RNA was prepared with RNAiso Plus (Takara; Beijing, China). The cDNA was obtained using the PrimeScript RT Reagent Kit with gDNA Eraser (Takara). qRT-PCR was conducted with SYBR Premix Ex Taq II (Takara) in the CFX96 Real-Time PCR Detection System (Bio-Rad Laboratories, CA, USA). Table S1 enlists the primer sequences. The relative gene expression was calculated by the 2^–ΔΔCT^ method.

### 16S rRNA gene sequencing of oral microbiota

Oral bacteria were obtained by oral swabs from the OVX and sham group rats. Then, *16S rRNA* gene sequencing of oral microbiota was performed by Majorbio company (Shanghai, China) [15]. Total DNA was extracted and purified. The Quant-iTTM PicoGreen reagent (Invitrogen, CA, USA) was applied to quantify the integrity of total DNA. The V3–V4 region of bacterial 16S rRNA was amplificated with the template of oral microbiota DNA samples and the *338F*/*806 R* primers (primer sequences are listed in Table S1). MiSeq 300PE (Illumina MiSeq System, CA, USA) was used to sequence the amplicon. Bioinformatics analysis was performed by Mothur and QIIME2.0 software.

### Cultivation of oral bacteria

Oral microbiota was obtained by oral swabs from the rats in bacteria from OVX group, bacteria from sham group, and PGF group. Then the oral microbiota was cultured on the blood agar plate in both aerobic and anaerobic conditions at 37°C for 48 h.

### Statistical analysis

All data were expressed as mean ± SEM and analyzed by GraphPad Prism 8 (GraphPad Software, CA, USA). For all experiments, a minimum sample size of 6 was used based on literature documentation of similar well-characterized experiments and using the ‘resource equation’ method [16–18]. Unpaired Student’s t-test was used to compare two independent groups. For multiple comparisons, one-way or two-way analysis of variance (ANOVA) followed by Tukey’s test was performed. A two-tailed Wilcoxon rank-sum test by R Project was conducted, for analyzing oral microbiota sequencing data. A *P*-value of < 0.05 was considered to be statistically significant.

## Results

### OVX rats display increased trabecular bone resorption in alveolar bone

**Figure 1.**
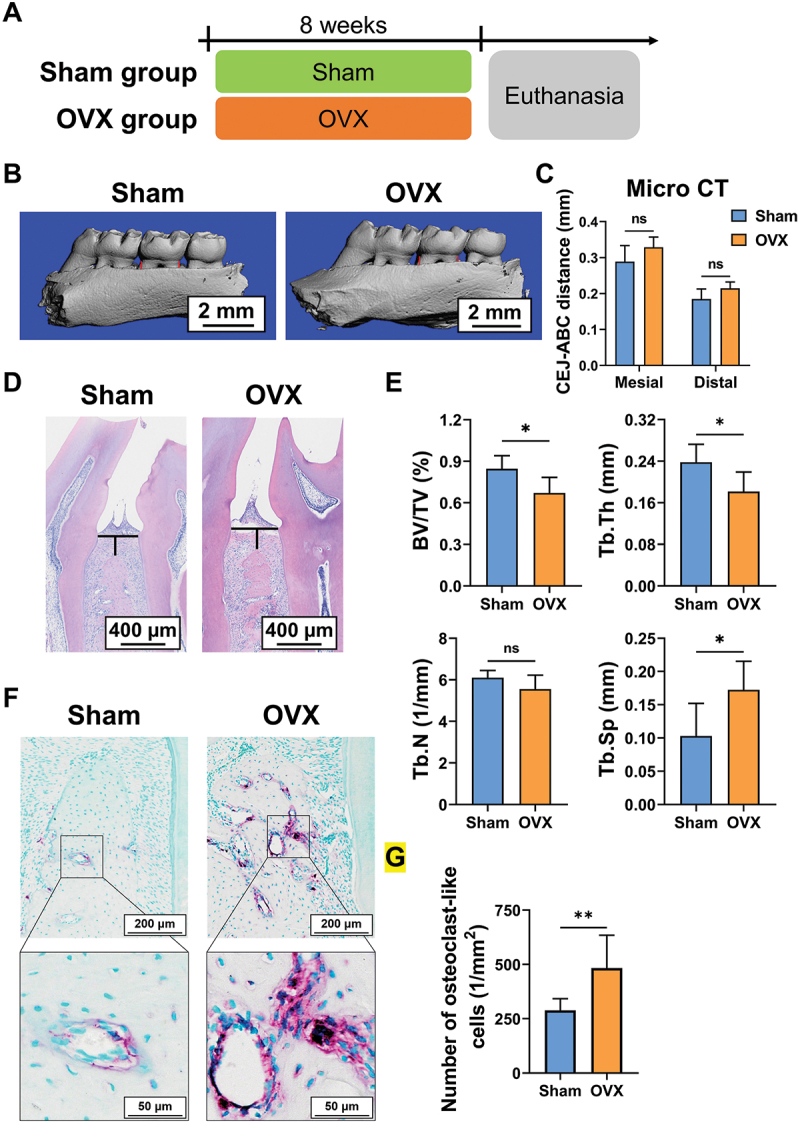
OVX rats display increased trabecular bone resorption in the alveolar bone. (A – G) the rats underwent bilateral ovariectomy or sham operation. (A) design of the experiment. (B) micro-CT reconstruction of the maxillary bone. The red line indicates the CEJ – ABC distance. (C) quantification of the CEJ – ABC distance by micro-CT. (D) H&E staining indicates CEJ – ABC distance at the interproximal sites between the first and second molar (black line). (E) quantitative analysis of trabecular bone parameters by micro-CT. (F) TRAP staining of the periodontal tissue. (G) quantification of osteoclast-like cells per bone surface. *p < 0.05, **p < 0.01, ns: not significant.

Previous studies have demonstrated that rats with OVX – periodontitis have more alveolar bone loss than sham-periodontitis rats [6]. Ligation of the molar would promote plaque accumulation and aggravate periodontal inflammation. Thus, to investigate the detrimental effects caused by OVX alone, the animal model was established by bilateral ovariectomy in the female rats without ligating the molar (Figure 1(A)). The OVX rats gained more weight than the sham rats (Figure S1A). The maxillary bones of rats were collected and used for micro-CT and histologic analysis. Micro-CT analysis revealed no significant difference in the CEJ – ABC distance between OVX and sham rats (Figure 1(B,C)). H&E staining revealed similar results (Figure 1(D) and Figure S1B). Then, the microarchitecture of the alveolar bone was further determined. The OVX rats showed significantly decreased BV/TV and Tb.Th and increased Tb.Sp (Figure 1(E)). Finally, TRAP staining showed OVX group rats had increased osteoclast-like cells than the sham group (Figure 1(F,G)).

### OVX rats have increased periodontal inflammation

**Figure 2.**
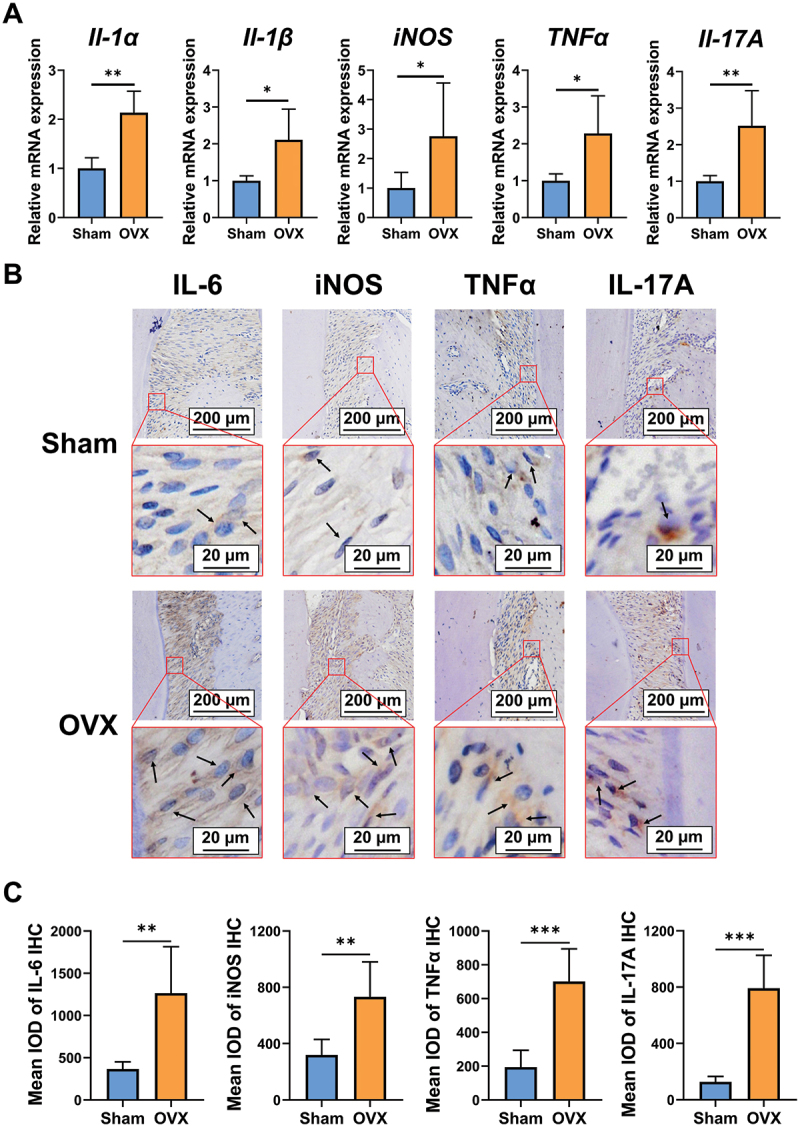
OVX rats have increased periodontal inflammation. (A – C) the rats underwent bilateral ovariectomy or sham operation. (A) gingival mRNA levels of *il-1α*, *il-1β*, *iNOS*, *TNFα*, and *il-17A*. (B) IHC staining of IL-6^+^, iNOS^+^, TNF*α*^+^, and IL-17A^+^ cells (black arrows) in the periodontal ligament tissue. (C) quantification of IL-6^+^, iNOS^+^, TNF*α*^+^, and IL-17A^+^ cells. *p < 0.05, **p < 0.01, ***p < 0.001.

Periodontitis is characterized by the inflammation of the supportive tissue surrounding teeth. Therefore, this study further investigated whether OVX alone would affect inflammation levels in the periodontal tissue. qRT-PCR illustrated that ovariectomy significantly increased the mRNA levels of *Il-1α*, *Il-1β*, *iNOS*, *TNFα*, and *Il-17A* in the gingival tissue of rats (Figure 2(A)). IHC staining was further conducted to determine the protein levels of inflammatory cytokines levels in the periodontal ligament tissue. Figure 2(B,C) demonstrate that OVX rats have increased IL-6, iNOS, TNF*α*, and IL-17A expressions.

### Oral microbiota plays an essential role in the influence of PMO on periodontitis

**Figure 3.**
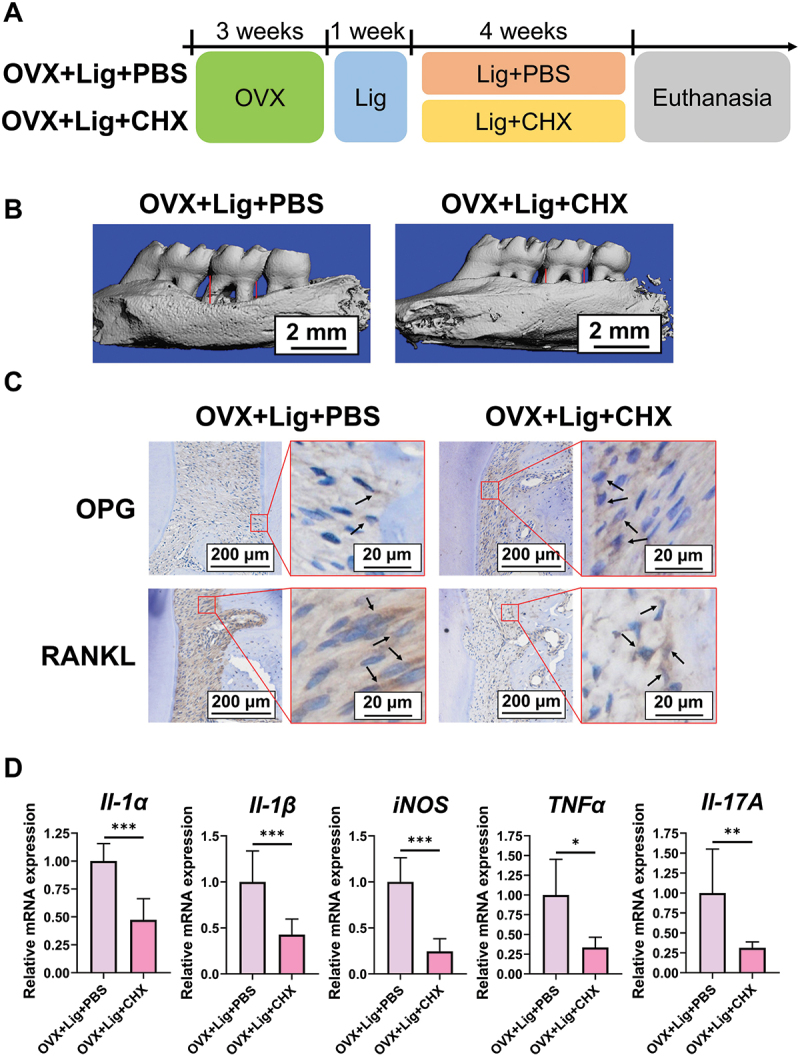
Oral microbiota plays a crucial role in the influence of PMO on periodontitis. (A – D) the rats underwent bilateral ovariectomy. Three weeks after the ovariectomy, the maxillary second molars of the rats were ligated for periodontitis. One week later, the OVX + lig + CHX group rats daily received the topical delivery of CHX, and the sutures were changed daily. In contrast, the OVX + lig + PBS group rats received the topical delivery of PBS daily for four weeks. (A) design of the experiment. (B) micro-CT reconstruction of the maxillary bone. The red line indicates the CEJ – ABC distance. (C) IHC staining of OPG^+^ and RANKL^+^ cells (black arrows) in the periodontal ligament tissue. (D) gingival mRNA levels of *il-1α*, *il-1β*, *iNOS*, *TNFα*, and *il-17A*. *p < 0.05, **p < 0.01, ***p < 0.001.

Apart from inflammation, oral microbiota is also a pivotal factor for periodontitis. However, whether oral microbiota participates in the influence of PMO on periodontitis remains unknown. There are two hypotheses. (1) PMO affects periodontitis by altering the periodontium’s inflammation level without the assistance of oral microbiota. (2) PMO affects periodontitis by inflammation and depends on oral microbiota. To test the involvement of oral microbiota in the impact of PMO on periodontitis, the clinically prescribed antimicrobial agent CHX was topically applied to the oral cavity of rats with OVX – periodontitis (Figure 3(A)). The administration of CHX significantly reduced alveolar bone loss (Figure 3(B), Figure S2A). OPG and RANKL are vital in the differentiation and maturation of osteoclasts [19]; hence, their expression levels in the periodontal tissue were investigated. The OVX + Lig + CHX group rats had increased OPG and decreased RANKL levels, compared with the OVX + Lig + PBS group rats (Figure 3(C)). Additionally, the depletion of the oral microbiota also decreased the gingival mRNA levels of *Il-1α*, *Il-1β*, *iNOS*, *TNFα*, and *Il-17A* (Figure 3(D)).

### PMO changes the oral microbiota composition

**Figure 4.**
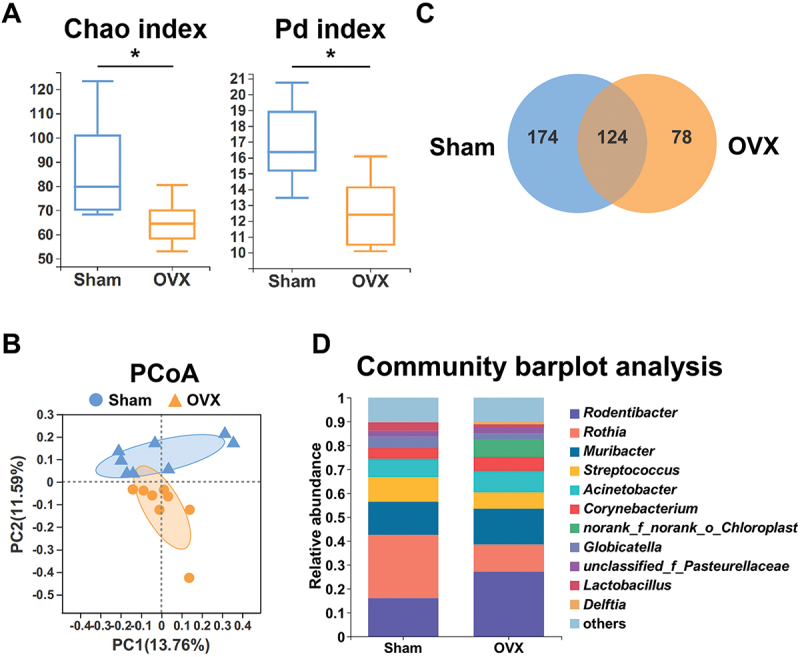
Ovariectomy alters the oral microbiota composition. (A – D) the rats underwent bilateral ovariectomy or sham operation. (A) the α-diversity of oral bacterial richness (Chao index) and phylogenetic diversity (Pd index). (B) the PCoA plot of the oral bacterial composition at the genus level. (C) the Venn diagram of oral bacteria at the genus level. (D) the taxonomic distribution of oral bacterial composition at the genus level.

Since oral microbiota is essential in the influence of PMO on periodontitis, whether PMO could affect the dynamic balance of oral bacteria to deteriorate periodontitis merits further investigation. Thus, we next explored the composition of the oral microbiota in OVX and sham rats. *16S rRNA* gene sequencing was conducted. The α-diversity analysis based on Chao and phylogenetic diversity (PD) indexes demonstrated that OVX significantly altered the richness and PD of the oral flora in rats (Figure 4(A)). Congruously, principal coordinate analysis (PCoA) showed significant differences in the oral microbiota composition between the OVX and sham rats (Figure 4(B)). The Venn diagram summarized the total number of unique and overlapping genera between the two groups (Figure 4(C)). Figure 4(D) displays the oral bacterial composition at the genus level of OVX and sham group rats. Genus *Rodentibacter*, a group of gram-negative coccobacilli, which could cause infection, was increased in OVX rats. In contrast, genus *Rothia*, gram-positive pleomorphic bacteria, was decreased.

### PMO increases the oral microbiota’s pathogenicity

**Figure 5.**
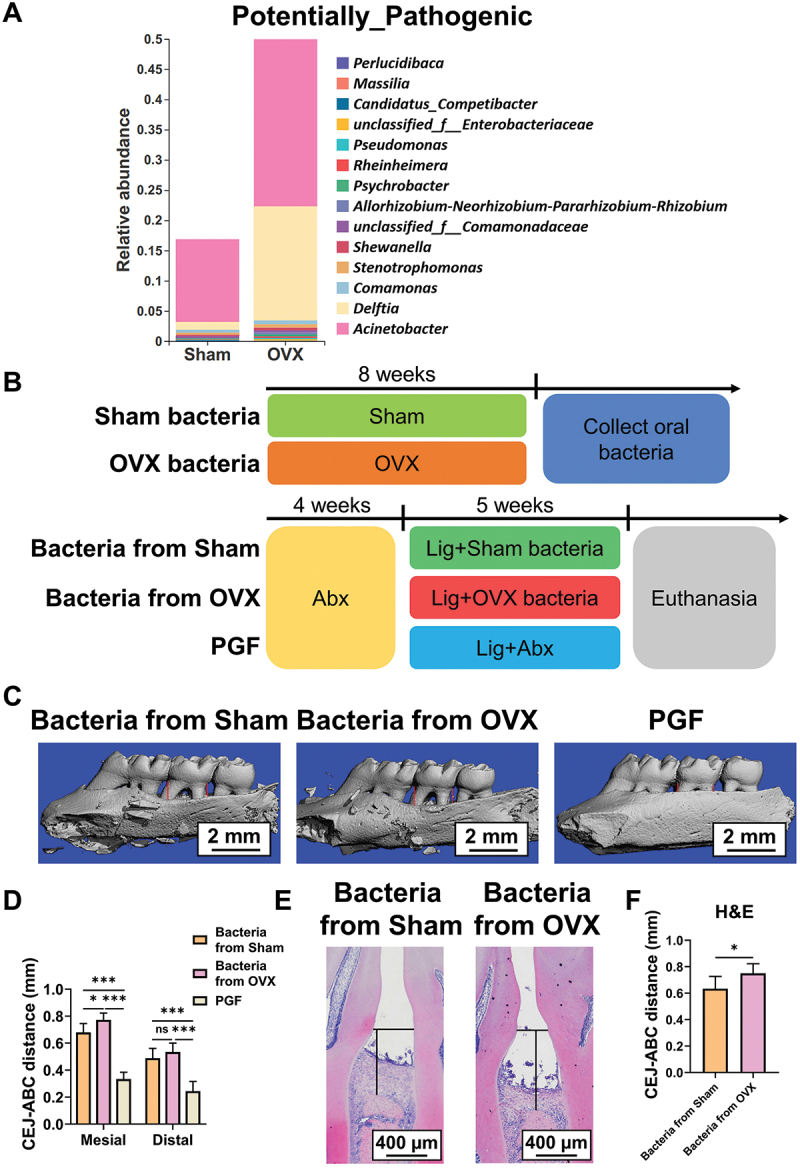
PMO increases the pathogenicity of the oral microbiota. (A) the BugBase analysis predicts the potentially pathogenic phenotype of the oral microbiota. The rats underwent bilateral ovariectomy or sham surgery. (B – F) oral microorganisms were collected from OVX and sham rats. The collected microorganisms were transferred to the PGF rats that ligated around the second molars. The PGF group rats were maintained on Abx in drinking water. Moreover, all the rats were raised for five weeks after receiving microorganism transplantation. (B) design of the experiment. (C) micro-CT reconstruction of maxillary bone. The red line indicates the CEJ – ABC distance. (D) quantification of mesial/distal CEJ – ABC distance by micro-CT. (E) H&E staining indicates CEJ – ABC distance at the interproximal sites between the first and second molar (black line). (F) quantification of the CEJ – ABC distance by H&E staining. *p < 0.05, ***p < 0.001, ns: not significant.

Since it has been proven that PMO would alter the composition of oral microbiota, we further investigated whether the pathogenicity of the oral microbiota in OVX rats was increased to promote alveolar bone loss. The BugBase analysis was applied to predict the potentially pathogenic phenotype of the oral microbiota. Figure 5A shows significantly higher proportions of potential pathogens in the oral microbiota of OVX rats. The oral microorganisms were transferred from the OVX and sham rats to PGF recipient animals with the maxillary second molars ligated to validate the results of the BugBase analysis (Figure 5B). No apparent alveolar bone resorption was observed in ligated PGF rats (Figure 5(C,D)). The micro-CT analysis showed that rats receiving bacteria from the OVX group exhibited an increased mesial alveolar bone loss in the second molars than the bacteria from the sham group rats (Figure 5(C,D)). The distal alveolar bone loss of the second molars in rats that received bacteria from the OVX group exhibited an increasing trend compared with rats from the sham group (Figure 5(C,D)). The H&E staining also showed similar results (Figure 5(E,F)).

## Discussion

Numerous studies have demonstrated that there is a positive correlation between PMO and periodontitis [5,20–22]. However, the underlying mechanism of this association is not clearly understood. Considering that inflammation and microorganism are essential for periodontitis, we examined the mechanisms of the influence of PMO on periodontitis from both aspects. This research demonstrates that OVX alone would enhance the periodontal inflammation and the pathogenicity of the oral microbiota, thus accelerating the progression of periodontitis.

Previous experimental studies have demonstrated that OVX-periodontitis rats endured more alveolar bone loss than sham-periodontitis rats [6,15]. However, in these investigations, the detrimental effects of PMO and ligature-induced alveolar bone loss were mixed. To determine the impact of PMO alone, we evaluated the alveolar bone resorption without establishing periodontitis. Our results showed that the OVX rats had increased trabecular bone resorption in the alveolar bone compared with sham rats. However, no significant difference was noted in the CEJ – ABC distance between OVX and sham rats, which may be explained by the time duration of the animal model. The alveolar bone loss was evaluated eight weeks after the OVX operation, which may not be enough to alter the CEJ – ABC distance. It is possible that the CEJ – ABC distance may increase if the modeling time were extended.

The inflammation plays a critical role in bone homeostasis and can serve as the mechanistic link between PMO and periodontitis [5]. The inflammatory cytokines produced during inflammatory response could affect the differentiation and activity of osteoblasts and osteoclasts. This study found that inflammatory cytokines in the gingival tissue and periodontal ligament tissue of OVX rats were increased, which could lead to increased bone resorption.

The association between oral microbiota and diseases is widely recognized. However, few studies have reported the alteration of the oral microbiota caused by PMO. We performed *16S rRNA* sequencing of oral microbiota in OVX and sham rats, which indicated significant differences in the oral microbiota composition between the two groups. *Rodentibacter* was the predominant genus in OVX rats and has a higher abundance in OVX rats than in sham rats. Genus *Rodentibacter* is a group of opportunistic pathogens that cause localized diseases with respect to favoring factors [23]. For example, some members of the genus *Rodentibacter* are related to lethal pneumonia in rodents [24]. Genus *Rodentibacter* was also one of the dominant genera in the pulp chamber of apical periodontitis rats [25]. However, there is little literature describing the role of *Rodentibacter* in periodontitis. We speculate that the abundance of *Rodentibacter* may also relate to the periodontal trabecular bone resorption in OVX rats; this hypothesis needs further confirmation. In addition, *Rothia* was the predominant genus in sham group rats. Barbagallo et al. discovered that *Rothia* was more prevalent in healthy sites than in periodontal sites [26]. In addition, the abundance of *Rothia* was found to be increased following periodontal therapy [27]. Therefore, the decreased abundance of *Rothia* in OVX rats may also link to the increased alveolar bone resorption.

The GF model is widely used to investigate specific microbiota transplantation’s effects on the host’s physiological and psychological functions [28], and the PGF model is commonly used to replace the GF model in many studies. In this study, we established the PGF model using large doses of broad-spectrum Abx to explore the pathogenicity of oral microbiota in OVX and sham rats. The PGF model is well-established and widely used in gut microbiota research; however, its use in the investigation of the effects of oral microbiota is still in its infancy. Li et al. showed that oral gavage of Abx exerts little impact on periodontal mature and dense biofilm in mice with ligature-induced periodontitis [12]. However, when Abx was administered in drinking water to mice before ligation, oral bacteria could be almost entirely depleted by Abx [13]. Thus, in this study, we dissolved Abx in drinking water and gave it ad libitum to rats. No bacterial growth was observed in the PGF group rats when culturing the oral sample on the blood plate after 5 weeks of ligation (Figure S3), indicating that the Abx treatment helped deplete oral microbiota.

The increased oral microbiota’s pathogenicity of OVX rats indicated that patients with PMO may be more susceptible to alterations in the oral microbiota toward dysbiosis in the occurrence and development of periodontitis. This result supports more frequent monitoring of the oral microbiota and more attention toward biofilm control in patients with PMO because the change of the oral microbiota in PMO patients could trigger or lead to more severe periodontitis.

However, there are limitations to note. First, to investigate whether oral microbiota was essential in the influence of PMO on periodontitis, CHX was used to deplete the oral microbiota, which is the most commonly used approach [29,30]. However, CHX could not eliminate all bacteria in the oral cavity [31]. Meanwhile, the multifilament of sutures would increase the surface area for bacterial colonization and let more bacteria be wicked into the suture tract [32]. Thus, to minimize the oral bacterial load, the ligatures were changed daily. In order not to interrupt the composition of the oral microbiota in the control group, we didn’t change the ligatures of the control rats. When changing the ligature of the OVX + Lig + CHX group rats, we tried our best to operate gently to avoid causing more damage than the control group, but the trauma cannot be completely avoided. If better strategies that could totally deplete the oral microbiota of periodontitis animals without changing the ligatures become available in the future, the effects of oral microbiota transplantation on the host’s health and diseases will be more transparent. In addition, there is a complex interaction between PMO, oral microbiota, and inflammation. PMO increases periodontal inflammation. Meanwhile, there is a shift in the oral microbiota in PMO rats. However, it is unclear whether inflammation or the shift of oral microbiota occurs earlier in the PMO rats. The first possibility is that PMO enhances periodontal inflammation, and then the increased periodontal inflammation causes a shift in oral microbiota. Another possibility is that PMO causes a pathogenic change in the oral microbiota, and then the shift of oral microbiota increases the periodontal inflammation. The specific mechanism is worthy of further investigation.

## Conclusion

In summary, this study’s findings show that PMO increases periodontal inflammation and oral microbiota’s pathogenicity to deteriorate periodontitis. They provide a better mechanistic understanding of how PMO increases the risk and severity of periodontitis. Moreover, the results also bolster the importance of more frequent monitoring of the oral microbiota and more attention toward biofilm control in patients with PMO.

## Supplementary Material

Supplementary Material.docx

## Data Availability

All raw reads have been deposited in the NCBI Sequence Read Archive (SRA) under accession number PRJNA909062.
