# Supplementary material for: Postmenopausal osteoporosis increases periodontal inflammation and the pathogenicity of the oral microbiota in a rat model
Source: J Oral Microbiol. 2025 Sep 1;17(1):2554381. doi: 10.1080/20002297.2025.2554381 (PMC12406332; doi:10.1080/20002297.2025.2554381)
Supplement: Supplementary Material.docx [file ZJOM_A_2554381_SM4370.docx]

**Title: Postmenopausal osteoporosis increases periodontal inflammation and the pathogenicity of the oral microbiota in a rat model**

**Supplementary material**


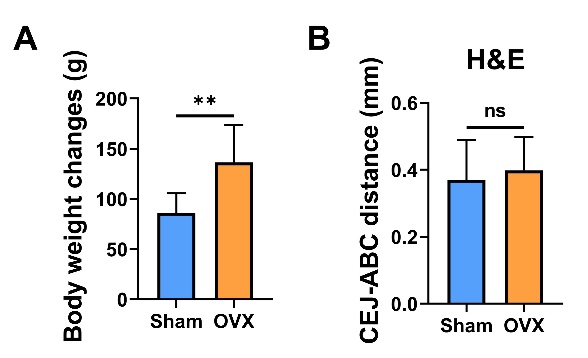


**Figure S1.** OVX rats display increased trabecular bone resorption in the alveolar bone. (A, B) The rats underwent bilateral ovariectomy or sham surgery. (A) Body weight changes of the OVX and sham rats. (B) Quantification of the CEJ–ABC distance by H&E staining. ** *P* < 0.01, ns: not significant.


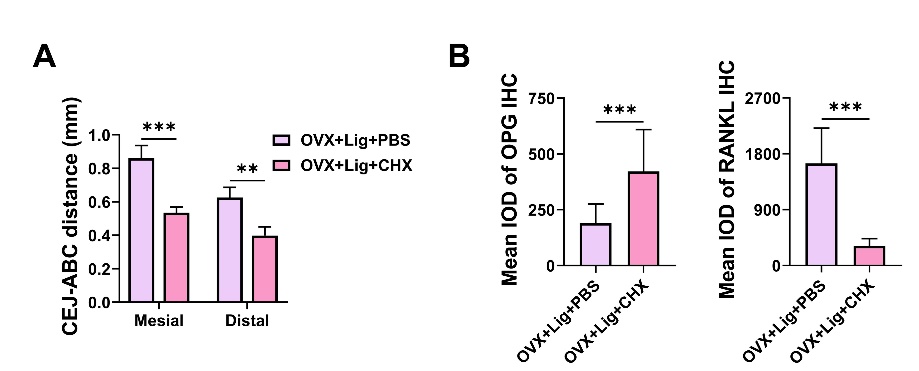


**Figure S2.** Oral microbiota plays an essential role in the influence of PMO on periodontitis. (A, B) The rats underwent bilateral ovariectomy. Three weeks after the ovariectomy, the maxillary second molars of the rats were ligated for periodontitis. One week later, the OVX + Lig + CHX group rats daily received the topical delivery of CHX, and the sutures were changed daily. In contrast, the OVX + Lig + PBS group rats received the topical delivery of PBS daily for four weeks. (A) Quantification of mesial/distal CEJ–ABC distance by micro-CT. (B) Quantification of OPG^+^ and RANKL^+^ cells in the periodontal ligament tissue. ** *P* < 0.01, *** *P* < 0.001.


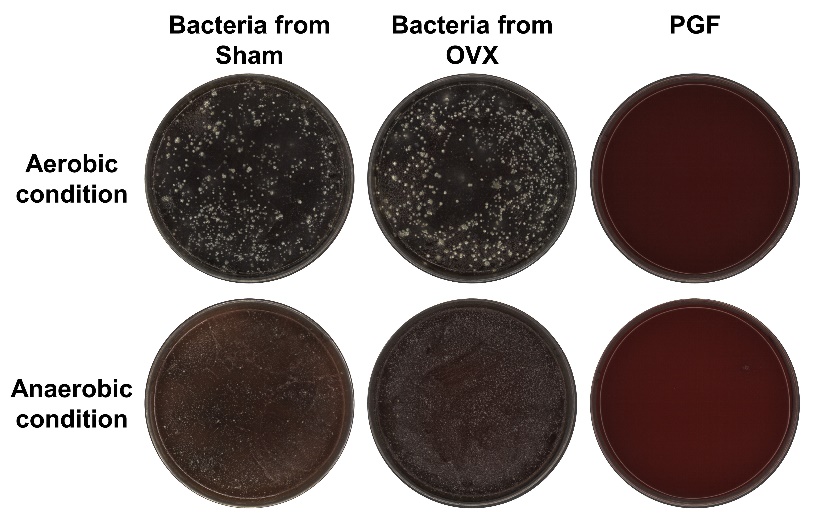


**Figure S3.** Effects of a broad-spectrum antibiotic cocktail (Abx) on the accumulation of oral bacteria in rats. Rats were treated with Abx for four weeks. Then rats received oral microbial transplant and were ligated for periodontitis for five weeks in the bacteria from sham group and the bacteria from OVX group rats. The PGF group rats were ligated and maintained on Abx in drinking water. The oral microbiota was collected and cultured on the blood plate after 5 weeks of ligation.

**Table S1. List of primers used in this study.**

| **mRNA/primer** | **Forward (5’-3’)** | **Reverse (5’-3’)** |
| --- | --- | --- |
| ***β-actin*** | CTGAGCTGCGTTTTACACCCT | CGCCTTCACCGTTCCAGTTT |
| ***Il-1α*** | GATCAGCACCTCACAGCTTCC | TAGAGTCGTCTCCTCCCGATG |
| ***Il-1β*** | GCTGTGGCAGCTACCTATGTCTTG | AGGTCGTCATCATCCCACGAG |
| ***iNOS*** | GAAACTTCTCAGCCACCTTGG | CCGTGGGGCTTGTAGTTGAC |
| ***TNFα*** | GGCGTGTTCATCCGTTCTC | CTTCAGCGTCTCGTGTGTTTCT |
| ***Il-17A*** | CTCATCCCTCAAAGTTCAGTGT | GCTAAGGGAGTTGAGGACTTTC |
| ***338F*/*806R*** | ACTCCTACGGGAGGCAGCAG | GGACTACHVGGGTWTCTAAT |
